# Oxymatrine Inhibits Renal Tubular EMT Induced by High Glucose via Upregulation of SnoN and Inhibition of TGF-β1/Smad Signaling Pathway

**DOI:** 10.1371/journal.pone.0151986

**Published:** 2016-03-24

**Authors:** Lirong Liu, Yuanyuan Wang, Rui Yan, Shuang Li, Mingjun Shi, Ying Xiao, Bing Guo

**Affiliations:** 1 Department of Clinical Hematology, Affiliated Hospital of Guizhou Medical University, Guiyang, Guizhou, China; 2 Department of Pathophysiology, Guizhou Medical University, Guiyang, Guizhou, China; 3 Department of Nephrology, Affiliated Hospital of Guizhou Medical University, Guiyang, Guizhou, China; Institute of Hepatology, Foundation for Liver Research, UNITED KINGDOM

## Abstract

Transforming growth factor-β1 (TGF-β1) signaling has been shown to play a critical role in the development of diabetic nephropathy (DN). The nuclear transcription co-repressor Ski-related novel protein N (SnoN) is an important negative regulator of TGF-β1/Smad signal transduction, and subsequent biological responses including tubule epithelial-mesenchymal transition (EMT), extracellular matrix accumulation and tubulointerstitial fibrosis. Oxymatrine (OM) is an alkaloid extracted from the Chinese herb Sophora japonica and has been demonstrated to prevent fibrosis. However, the anti-fibrosis effect of OM in DN is still unclear. In this study, we cultured normal rat renal tubular epithelial cells (NRK52Es) in high glucose and high glucose plus OM, and detected the expression of E-cadherin, α-SMA, FN, TGF-β1, SnoN, Arkadia, p-Smad2 and p-Smad3 and poly-ubiquitination of SnoN. The results showed that E-cadherin and SnoN expression in NRK52Es decreased significantly, but poly-ubiquitination of SnoN, TGF-β1, α-SMA, FN, Arkadia, p-Smad2 and p-Smad3 expression significantly increased due to high glucose stimulation, which could be almost completely reversed by OM, suggesting that OM may alleviate EMT induced by high glucose via upregulating SnoN expression and inhibiting TGF-β1/Smad signaling pathway activation. Hence, OM could be a novel therapeutic for DN.

## Introduction

Diabetic nephropathy (DN) is one of the most common and serious microvascular complications of diabetes mellitus (DM) [1]. Pathological characteristics of DN are basement membrane thickening, renal tubal epithelial-mesenchymal transition (EMT), extracellular matrix (ECM) accumulation, glomerulosclerosis and tubulointerstitial fibrosis (TIF), eventually leading to irreversible renal damage [2–5]. The pathogenesis of DN remains unclear and the current treatments have limited effectiveness [6]. Thus, in order to develop new effective therapeutic measures for DN, it is necessary to further investigate its molecular mechanisms.

EMT is a process by which epithelial cells lose their orientation and cell-cell contact, and acquire migratory and invasive properties of mesenchymal cells. Transforming growth factor-β1 (TGF-β1) is known as a key mediator of fibrogenesis, which induces and regulates the EMT, ECM accumulation and TIF progression by the TGF-β1/Smad signaling pathway in DN [7–10]. Nuclear transcription co-repressor Ski-related novel protein N (SnoN) is one of the most important factors that negatively regulate the TGF-β1/Smad signaling pathway. SnoN associates with Smads to block the transduction of TGF-β1 signaling and inhibit the transcriptional activation of TGF-β1 responsive genes [11, 12]. In order to counteract inhibition of transcription by SnoN, TGF-β1/Smad signaling induces the degradation of SnoN by the ubiquitin-proteasome pathway (UUP) [13, 14]. Arkadia is a member of the RING finger ubiquitin ligase superfamily that promotes activation of the TGF-β1 signaling pathway. Upon activation of TGF-β1 signaling, Arkadia binds to phosphorylated Smad2/3 (p-Smad2/3) and induces degradation of Smad7 and SnoN/Ski, enabling transcription of TGF-β1 target genes [15–19].

**Fig 1:**
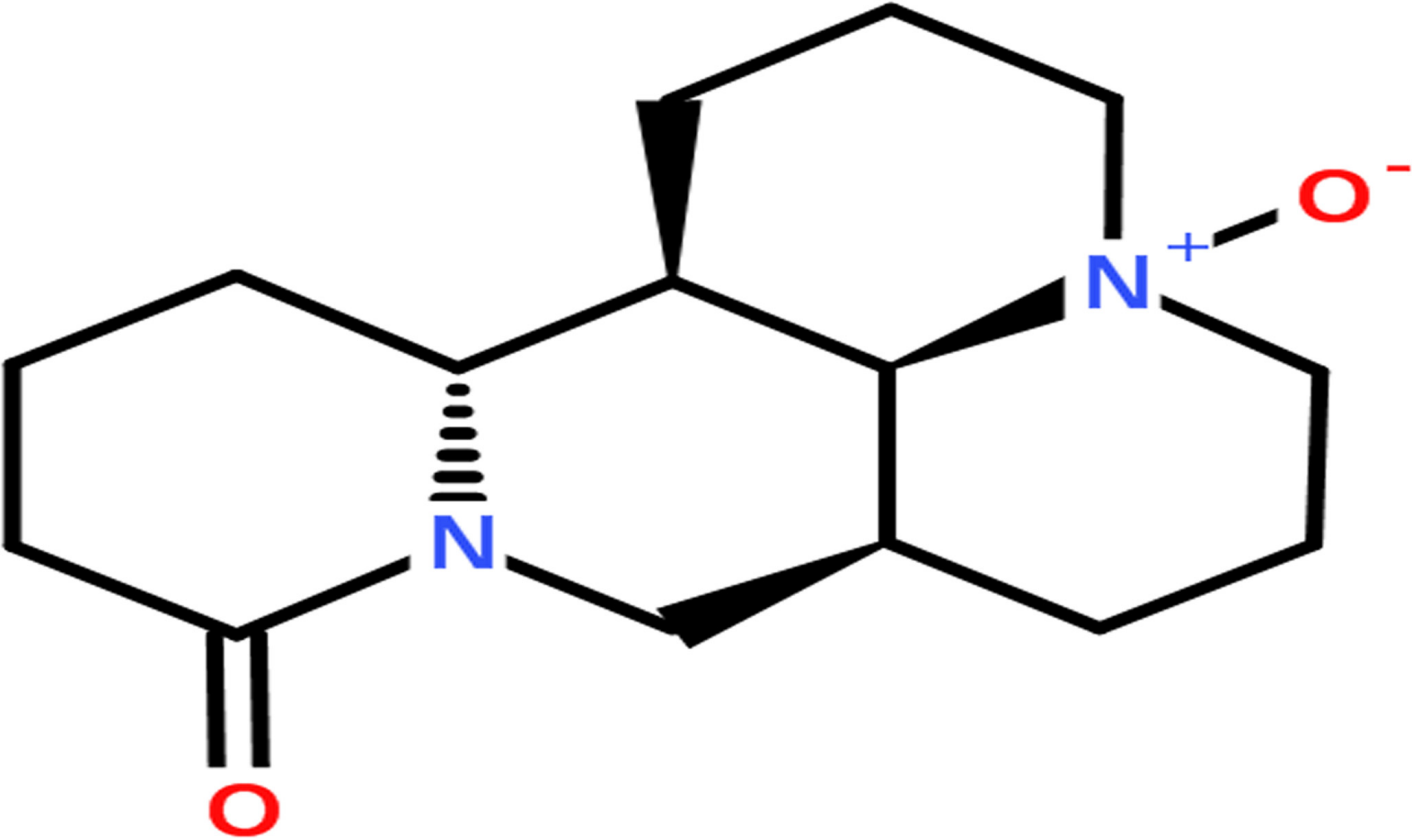
The chemical structure of OM.

Oxymatrine (OM) is an herbal product derived from the root of Sophora flavescens Ait. OM has a tetracyclic quinolizine structure (Fig 1), and its molecular formula is C15H24N2O. OM is reported to have anti-inflammatory, anti-oxidative, anti-viral, anti-fibrotic and immunological regulation effects [20, 21]. In recent years, OM has been used in China for the treatment of various human illnesses such as hepatitis B infections and liver fibrosis [22–25]. Previous studies have demonstrated that OM had an anti-fibrotic effect on liver fibrosis, pulmonary fibrosis, myocardial fibrosis and skin scar tissue fibrosis via inhibition of the TGF-β/Smad signaling pathway [26–31]. However, the molecular mechanism underlying its pharmacological effects and whether OM can protect against renal fibrosis in DN remains unclear.

Thus, this study aimed to investigate whether OM inhibited EMT induced by high glucose in normal rat renal tubular epithelial cells (NRK52Es) in vitro and identify the potential molecular mechanisms in order to provide important experimental evidence to support the use of OM in the prevention and treatment of DN.

## Materials and Methods

### Cell Culture, Treatment, and Transient Transfection

The well-characterized NRK52Es were provided by Professor Limin Lu, Fudan University, China, who purchased the cells from the Cell Bank of Chinese Academy of Sciences (Shanghai, China). NRK52Es were cultured in low-glucose Dulbecco's modified eagle medium (DMEM) with 10% fetal bovine serum (Hyclone, USA) at 37°C and 5% CO2. The cells were randomly divided into three groups: normal glucose control group (NG group, with medium containing 5.5 mmol/L glucose), high glucose treatment group (HG group, with medium containing 25 mmol/L glucose), and OM therapy group (HM group, with medium containing 25 mmol/L glucose+0.50mg/ml OM). OM was purchased from the China National Institute of Control of Pharmaceutical and Biological Products (Batch Number: 110780–201007). Cells in each group were cultured for 48 hours (h). For SnoN degradation assay, NRK52Es were transiently transfected with pFlag-SnoN expression vector and then treated for 48h (data not shown). Transient transfection of NRK52Es was transfected by Lipofectamine 2000 according to the instructions specified by the manufacturer (Invitrogen).

### Immunofluorescence–laser scanning confocal microscopy (IF-LSCM)

NREK52Es were seeded into a 6-well plate on coverslips at a density of 1× 10^5^ cells/well and treated as above. After 48h, the cells were washed with PBS, fixed with 4% paraformaldehyde, blocked with goat serum and incubated overnight at 4°C with E-cadherin antibody (rabbit, 1:100, Santa Cruz, USA) and α-SMA antibody (mouse, 1:100, Sigma-Aldrich, USA). Then the cells were incubated for 15 minutes with FITC-labeled secondary antibody (1:50, Boster, Wuhan, China) and TRITC-labeled secondary antibody (1:50, Boster, Wuhan, China), respectively. Finally, the cells were incubated with DAPI (Boster, Wuhan, China) for 10 minutes. Accordingly, the cellular E-cadherin and α-SMA expression was quantitatively analyzed by laser scanning confocal microscope (Olympus FV1000, Olympus, Japan).

### Western blotting

Total proteins were extracted by lysing the cells in RIPA buffer (Kaiji, Shanghai, China), and protein concentration was determined by Bradford assay. Whole cell extracts were boiled in SDS sample buffer [125 mmol/L Tris-HCl (pH 6.8), 4% SDS, 20% glycerol, 100 mmol/L 2-mercaptoethanol and 0.02% bromophenol blue], and denatured proteins were separated by SDS-PAGE. The proteins were transferred onto a PVDF membrane (Millipore, Billerica, USA) and then the membranes were blocked in TBS-T buffer containing 5% non-fat dry milk, and probed with primary E-cadherin antibody (rabbit, 1:400, Santa Cruz, USA), α-SMA antibody (mouse, 1:400, Sigma-Aldrich, USA), FN antibody (goat, 1:150, Santa Cruz, USA), TGF-β1 antibody (mouse, 1:200, Santa Cruz, USA), ubiquitin antibody (mouse, 1:700, Santa Cruz, USA), SnoN antibody (goat, 1:300, Santa Cruz, USA), Arkadia antibody (rabbit, 1:300, Santa Cruz, USA), β-actin antibody (mouse, 1:400, Santa Cruz, USA), Phospho-Smad2 (p-Smad2) antibody (Ser465/467) and Phospho-Smad3 (p-Smad3) antibody (Ser423/425) (rabbit, 1:150, Cell Signaling Technology, USA), respectively, in blocking buffer at 4°C overnight. Blots were washed with TBS-T buffer and then incubated with horseradish peroxidase-conjugated secondary antibodies (1:5000, Santa Cruz, USA) at room temperature for 60 minutes. Finally, the protein bands were detected using the enhanced chemiluminescence system and ECL Hyperfilm (Amersham, England) and exposure to X-ray film. Band intensities were quantified using Bio-Rad gel imaging system (BIO-RAD, Hercules, USA) and Quantity one 4.6 software (Bio-Rad, USA). Anti-β-actin was used as internal control.

### RNA extraction and quantitative real-time PCR analysis

Total RNA was extracted using Trizol reagent (Invitrogen, Carlsbad, USA) according to the manufacturer’s protocol. First-strand cDNA was synthesized using Takara RNA PCR kit (Baoshengwu, Dalian, China) according to the manufacturer’s instructions. Gene expression levels were measured by real-time RT-PCR using SYBR Select Master Mix (BIO-RAD, Hercules, USA), and analyzed on a CFX 96^TM^ Connect Real-Time system (BIO-RAD, Hercules, USA). Primers for each gene were designed by DNA man software and synthesized by Generay Biotech Co., Ltd. (Shanghai, China). Target gene expression levels in each sample were subsequently normalized to the level of β-actin mRNA. Primers used were as follows:

TGF-β: Forward primer 5′ - GAAAGCCCTGTATTCCGTCTCC—3′, Reverse primer 5′ - GCAACAATTCCTGGCGTTACCT-3′.

SnoN: Forward primer 5′ - GCTTTACTGCGGCCACGAACTT—3′, Reverse primer 5′ - AGGGAGCGTCGGGCTGAACATA—3′.

Arkadia: Forward primer 5′ - CCTCACATCCGTTACATTTCTT—3′, Reverse primer 5′ - CTCCACGATTGACATTTCCTA—3′.

β-actin: Forward primer 5′ - ACCACCATGTACCCAGGCAT—3′, Reverse primer 5′ - CCGGACTCATCGTACTCCTG—3′.

### Immunoprecipitation

According to the manufacture’s instructions and as described in Ref. [32], Cells were lysed for 30 min on ice in lysis buffer (1% Triton X-100, 50 mM TrisHCl, pH7.5, 1 mM EDTA, 150 mM NaCl, 10% glycerol and protease inhibitors). The lysates were centrifuged at 15000 g for 15 min at 4°C and the supernatant was collected. Anti-Flag antibody (mouse, Sigma, USA) was added to the supernatant and incubated for 3 h with rotation at 4°C. Then, the immunecomplex was precipitated with protein A/G-sepharose beads (Santa Cruz, USA) and used for SnoN ubiquitination assay.

### In vitro SnoN Ubiquitination Assay

SnoN ubiquitination activity in NRK52Es was detected by an in vitro assay using exogenous Flag-tagged SnoN protein as substrate, as described elsewhere [33,34]. NRK52Es were transfected with Flag-tagged SnoN expression vector. Cell lysates from NG, HG or HM group NRK52Es (100 μg) were incubated with 6 μl of immunoprecipitated Flag-tagged SnoN protein and 100 μl ubiquitination mixture [10 mM Tris-HCl pH 7.6 and 2 mM MgCl_2_, 2 mM dithiothreitol, 100 μM ubiquitin aldehyde, 10 mM phosphocreatinine, 0.28 units/ml phospho-creatinine kinase, 1× protease inhibitor mix, 1× proteasome inhibitormix (Enzo Life Sciences, USA), 1 μM E1 enzyme (Ube1, Enzo Life Sciences, USA), 10 μM E2 enzyme (UbcH5b, Enzo Life Sciences, USA), 1 μM Hdm2 (Enzo Life Sciences, USA), and 10 μM peptide] for 1.5 h at 37°C. After incubation, the reactions were boiled in SDS sample buffer for 5 min, followed by Western blotting with anti-ubiquitin antibody.

### Statistical analysis

Each experiment was performed at least in triplicate. Data are expressed as mean ± SD. Statistical analyses were performed using SPSS 19.0 statistics software(SPSS, Chicago, IL). One-way ANOVA and Student’s Newman-Keuls test for comparisons were used to determine differences between control and experimental groups. Student's t-test was performed for paired samples. p < 0.05 was considered statistically significant.

## Results

### Effect of OM on high glucose-induced renal tubular EMT

**Fig 2:**
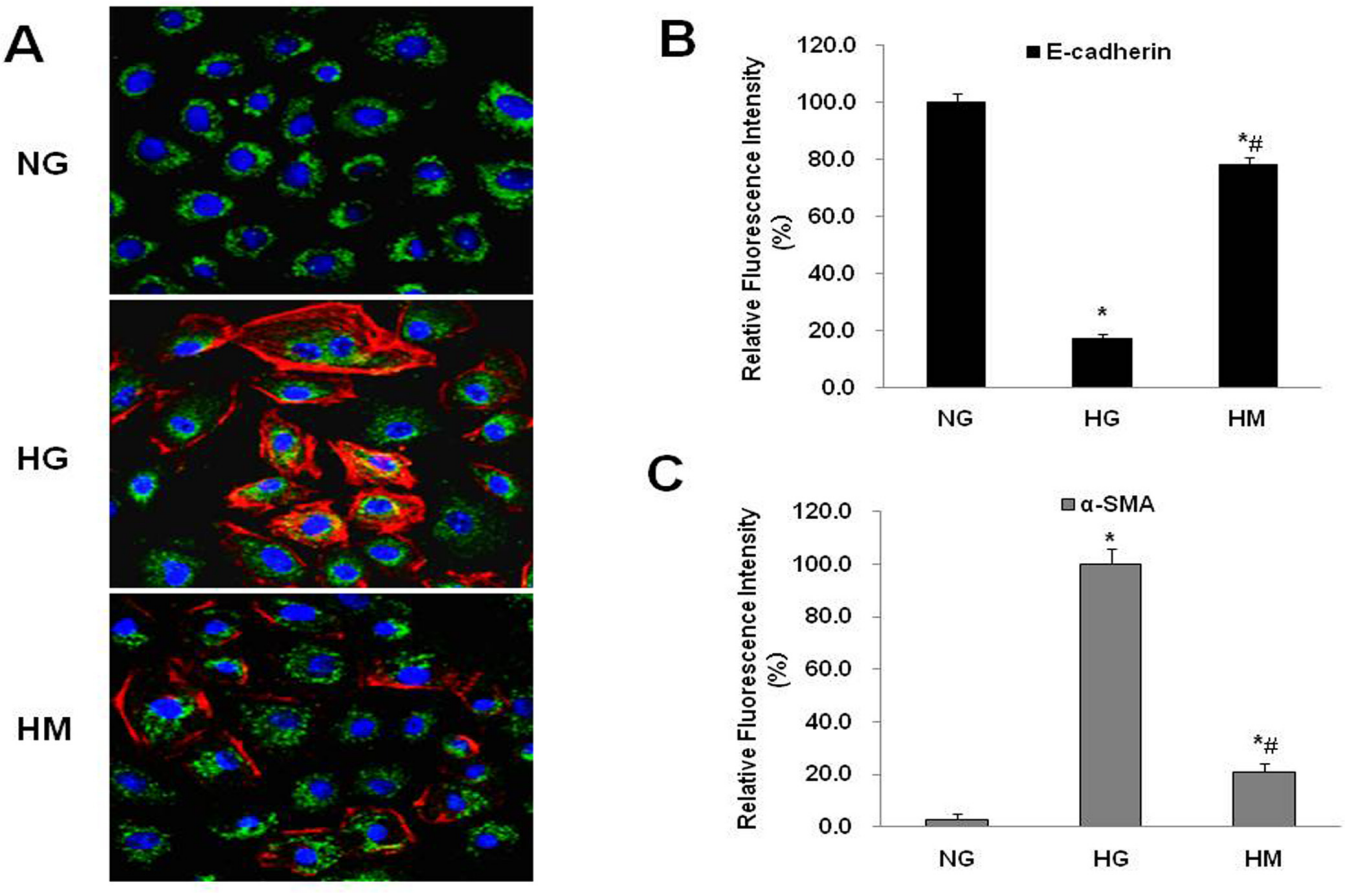
Effect of OM on E-cadherin and α-SMA expression by IF-LSCM. **A,** Triple-labeled LSCM images for E-cadherin (green), α-SMA (red) and nucleus (blue) of NRK52Es. The merged fluorescent images of E-cadherin, α-SMA and nucleus showed that E-cadherin (green) was distributed throughout the cytoplasm and α-SMA (red) was most prominent at the cytoplasmic membrane. The images of NG, HG and HM are at the same magnification. B, Relative Fluorescence Intensity (%) analysis demonstrated that the expression of E-cadherin was significantly decreased in the HG group as compared to the NG group (*P<0.05), but as compared to the HG group, the expression of E-cadherin was significantly increased in the HM group (#P<0.05). C, Relative Fluorescence Intensity (%) analysis demonstrated that the α-SMA expression was markedly increased in the HG group as compared to the NG group (*P<0.05), but as compared to the HG group, the expression of α-SMA was markedly decreased in the HM group (#P<0.05). Notes: NG, normal glucose control group; HG, high glucose group; HM, high glucose + OM group.

**Fig 3:**
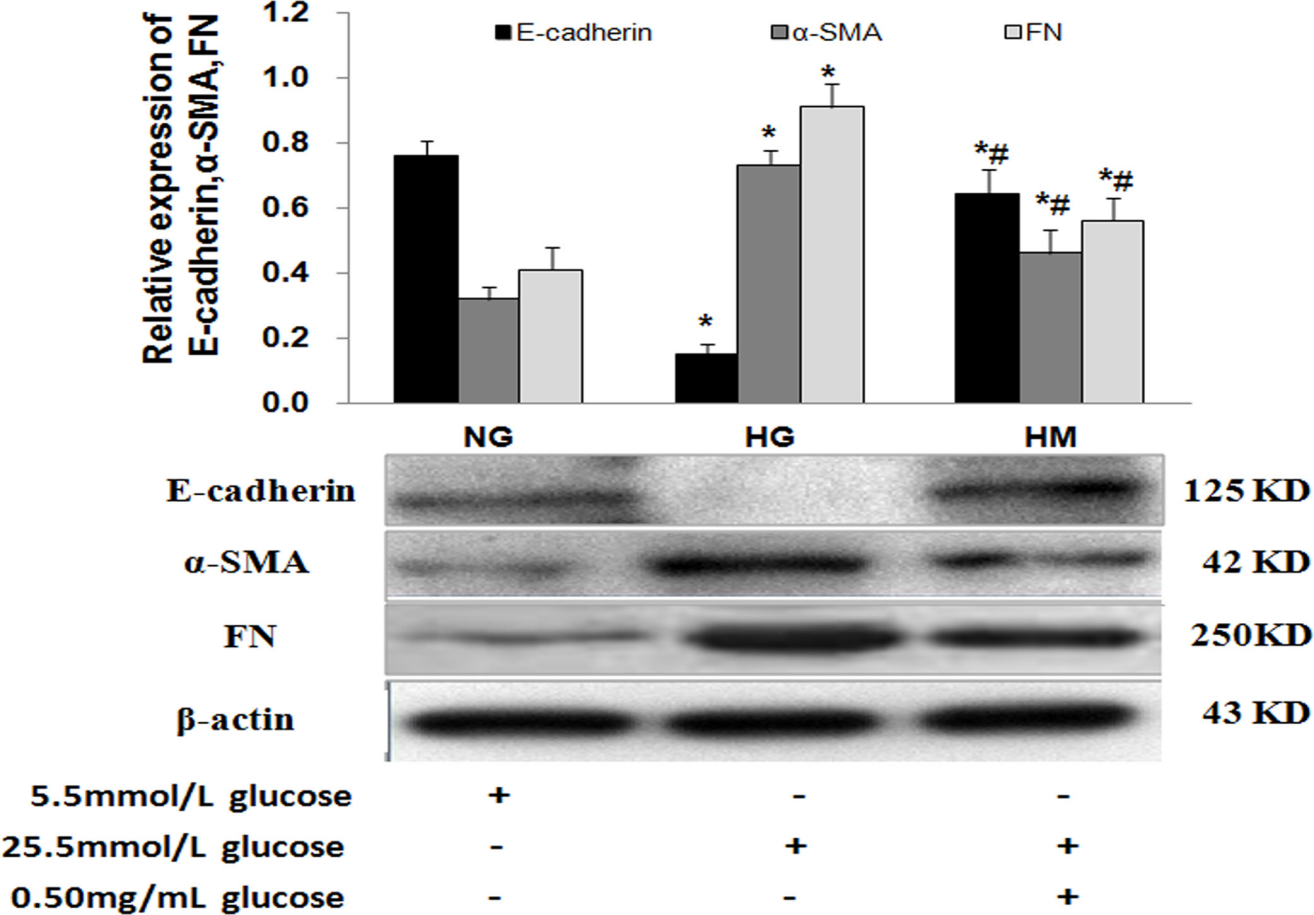
Effect of OM on the expression of E-cadherin, α-SMA and FN proteins by western blotting in each NRK52E group. The expression level of E-cadherin was significantly decreased whereas α-SMA and FN were markedly increased in the HG group as compared to the NG group (*P<0.05). The expression of E-cadherin was significantly increased while α-SMA and FN were significantly decreased in the HM group as compared to the HG group (#P<0.05).

To evaluate the effect of OM on NRK52Es, we have performed several experiments under normal glucose and high glucose conditions, including MTT assay, dose-dependent and time-dependent trials, and confirmed that a dose of 0.50ug/ul for 48h was unlikely to exert significant toxic effect on cells but had significant effects on key indicators [35]. Therefore, 0.50 mg/ml OM was used in the subsequent tests. To determine whether OM affected related EMT marker expression levels, E-cadherin, a marker for epithelial cells, and α-SMA, a marker for mesenchymal cells, were examined by IF-LSCM and western blotting, which showed that E-cadherin was significantly reduced and α-SMA was markedly increased in the HG group as compared to the NG group. After the cells were treated with 0.50mg/mL OM in high glucose condition, OM obviously upregulated E-cadherin expression and downregulated α-SMA expression (Figs 2A, 2B, 2C and 3). Taken together, these results demonstrated that OM could reverse the EMT induced by high glucose.

### Effect of OM on fibronectin (FN) in NRK52Es stimulated by high glucose

One key feature of DN was the accumulation of ECM proteins such as FN [36]. EMT mainly led to the deposition of ECM and renal fibrosis. To confirm whether OM affected ECM expression, we examined the expression of FN by western blotting, which showed that FN was significantly increased in the HG group as compared to the NG group. After the cells were treated with 0.50mg/mL OM in high glucose condition, the expression of FN was significantly decreased in the HM group as compared to the HG group (Fig 3). The results suggested that OM can alleviate the excessive deposition of FN induced by high glucose.

### Effect of OM on the expression of TGF-β1 and SnoN in NRK52Es stimulated by high glucose

**Fig 4:**
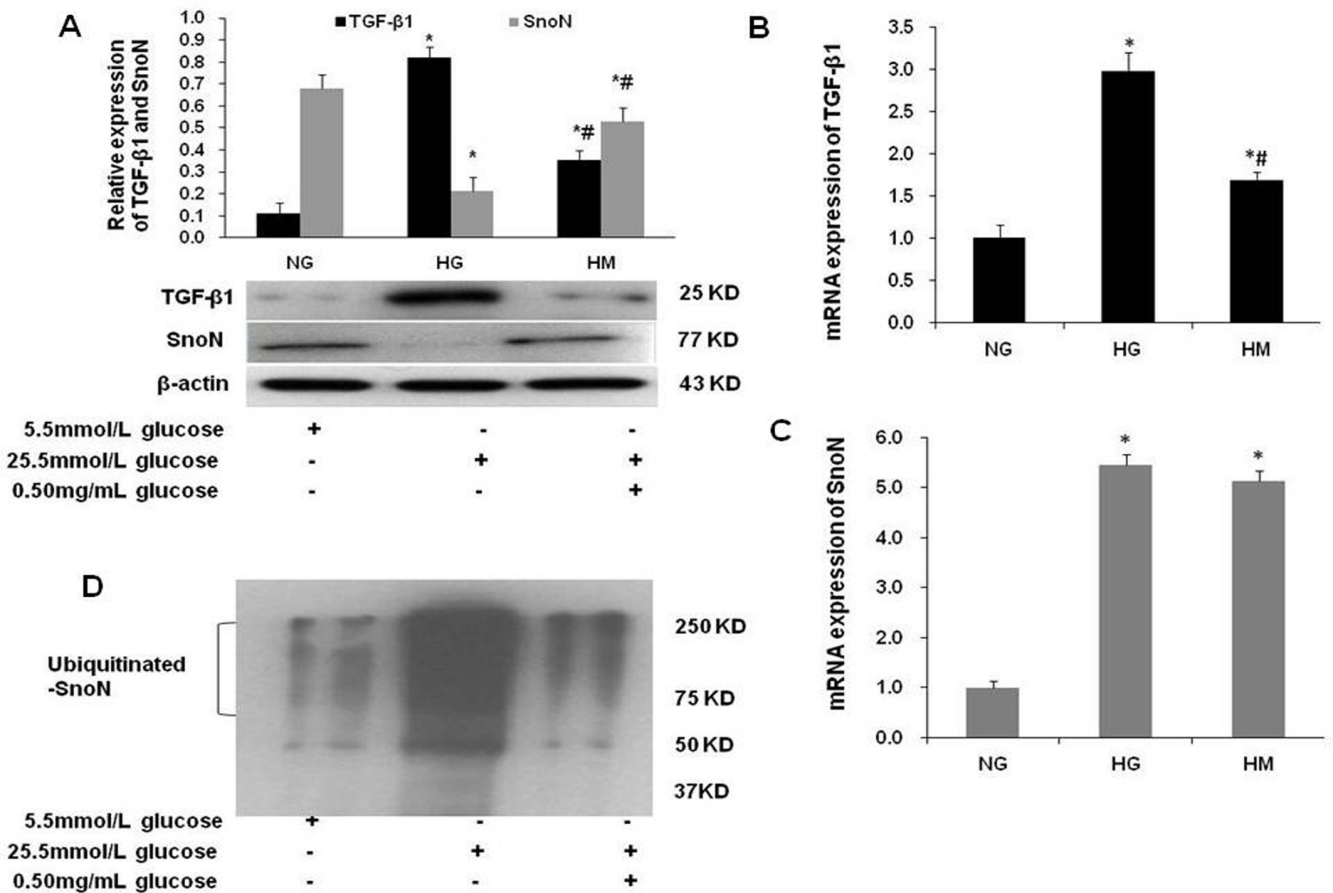
Effect of OM on TGF-β1 and SnoN expression by western blotting, real-time PCR and in vitro SnoN ubiquitination assay. **A,** Western blotting demonstrated that the expression level of TGF-β1 protein was markedly increased and SnoN was significantly decreased in the HG group as compared to the NG group (*P<0.05). The expression of TGF-β1 protein was significantly decreased while SnoN protein was significantly increased in the HM group as compared to the HG group (#P<0.05). **B,** Real-time PCR demonstrated that the mRNA expression level of TGF-β1 was consistent with its protein expression level in each group (*P < 0.05, #P<0.05). **C,** Real-time PCR demonstrated that the mRNA expression level of SnoN mRNA expression level was consistent with its protein expression level in the NG and HM groups (*P<0.05), whereas SnoN mRNA expression was significantly increased as compared to its protein level in the HG group (*P<0.05). **D,** In vitro SnoN ubiquitination assay demonstrated that SnoN was intensively poly-ubiquitinated in the HG group. The poly-ubiquitination of SnoN was significantly decreased in the HM group compared with the HG group.

The TGF-β1/smads signaling pathway plays an important role in EMT. SnoN inhibited TGF-β1/smads signaling pathway in DN, suggesting that SnoN has an antagonistic effect on EMT [37]. To determine whether OM affects the TGF-β1/smad signaling pathway in NRK52Es stimulated by high glucose and investigate the probable molecular mechanisms, we used western blotting and real-time PCR to examine the protein and mRNA expression of TGF-β1 and SnoN. Western blotting showed that the protein level of TGF-β1 was significantly increased and SnoN was significantly decreased in the HG group as compared to the NG group. Meanwhile, the protein expression of TGF-β1 and SnoN was significantly decreased in the HM group as compared to the HG group (Fig 4A). Real-time PCR results showed that TGF-β1 and SnoN mRNA levels were remarkably increased in the HG group as compared to the NG group. In the HM group, OM could decrease TGF-β1 mRNA expression induced by high glucose but did not affect the SnoN mRNA level as compared to the HG group (Fig 4B and 4C). Interestingly, the protein and mRNA levels of SnoN were in conflict in high glucose stimulated NRK52Es, which implied that high glucose-induced downregulation of SnoN expression was not caused primarily by an alteration at gene transcriptional level but probably resulted from an enhanced protein ubiquitin-dependent degradation. Taken together, these results demonstrated that OM could reverse the EMT induced by high glucose via downregulating TGF-β1 expression and upregulating SnoN expression.

To determine whether high glucose promotes SnoN degradation via a ubiquitination- and proteasome-dependent pathway and whether OM upregulates SnoN expression via attenuating high glucose-induced ubiquitin-dependent degradation of SnoN protein in NRK52Es, we performed an in vitro SnoN ubiquitination assay. The results showed that smeared band of the poly-ubiquitinated SnoN was markedly increased in the HG group as compared to the NG group. Meanwhile, the poly-ubiquitination of SnoN was significantly decreased in the HM group as compared to the HG group, suggesting that OM attenuates the high glucose-induced ubiquitin-dependent degradation of SnoN protein (Fig 4D).

### Effect of OM on the expression of Arkadia, p-Smad2 and p-Smad3 in NRK52Es stimulated by high glucose

**Fig 5:**
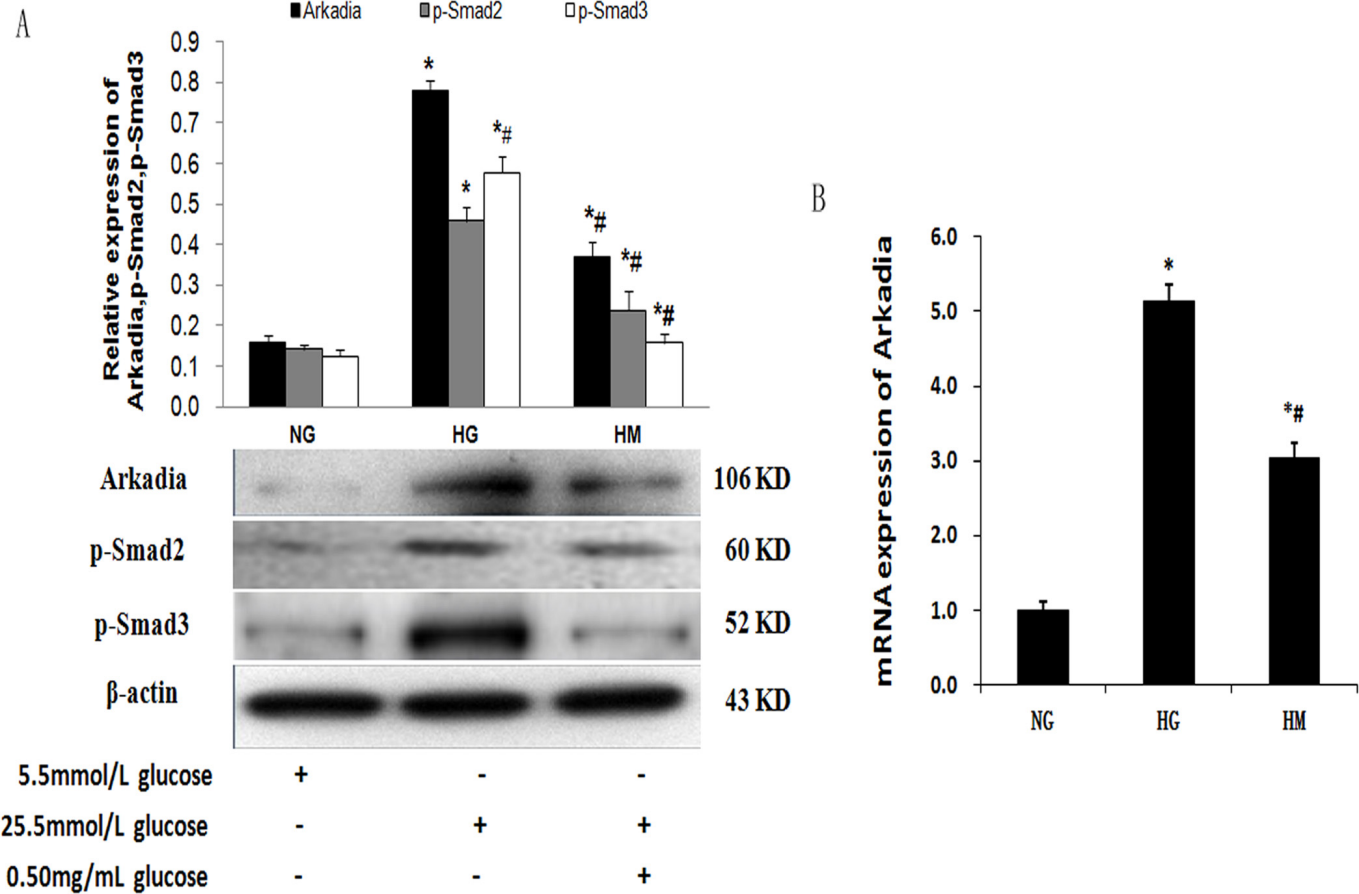
Effect of OM on the expression of Arkadia, p-Smad2 and p-Smad3 by western blotting and real-time PCR. **A,** Western blotting demonstrated that the protein expression levels of Arkadia, p-Smad2 and p-Smad3 were markedly increased in the HG group as compared to the NG group (*P<0.05) whereas, the expression of these proteins was significantly decreased in the HM group as compared to the HG group (#P<0.05). **B,** Real-time PCR demonstrated that the mRNA expression level of Arkadia was consistent with its protein expression level in each group (*P<0.05, #P<0.05).

Previous studies have found that Arkadia associates with SnoN proteins in their free forms, as well as when they are bound to Smad2/3 proteins [38–40]. There was no significant difference between the HG and HM groups in SnoN mRNA expression, which suggested that high glucose led to decreased SnoN expression perhaps through the ubiquitin-proteasome pathway (UPP) and OM upregulated SnoN expression perhaps by inhibiting ubiquitin ligase expression. To further investigate the molecular mechanisms involved in OM-inhibited EMT induced by high glucose, we determined whether OM upregulated SnoN expression by inhibiting Arkadia-mediated degradation of SnoN. Western blotting showed that OM could reverse high glucose induced high expression of Arkadia, p-Smad2 and p-Smad3 (Fig 5A) The results of real-time PCR showed that Arkadia was increased in the HG group as compared to the NG group, and OM significantly decreased Arkadia mRNA in the HM group as compared to the HG group (Fig 5B). Moreover, these results suggested that OM could inhibit the increase of Arkadia, p-Smad2 and p-Smad3 induced by high glucose and inhibit Arkadia-mediated degradation of SnoN, resulting in the increase of SnoN expression.

## Discussion

Tubulointerstitial fibrosis (TIF) is the principal route leading to end-stage renal failure with the development of DN, and is also a key factor to determine the extent of renal function deterioration. TGF-β1, as a fundamental mediator of fibrogenesis, plays a critical role in the renal tubular EMT process by a Smad-dependent pathway, resulting in TIF [41, 42]. Smad2/3 proteins are phosphorylated by TGF-β1 receptor and mediate the intracellular signal transduction of TGF-β1. Nuclear transcriptional co-repressor SnoN is recognized as an important negative regulating factor of TGF-β1/Smad signaling pathway, which could inhibit the activation of TGF-β1 target genes and interfere with the biological effects of TGF-β1/Smad signaling pathway by interacting with the downstream Smad proteins [43–45]. Our previous studies and others [36, 46, 47] have demonstrated that the expression of TGF-β1 significantly increased, while the expression of SnoN significantly decreased in kidney tissues from DN rats and renal tubular epithelial cells cultured in high glucose, which resulted in the persistent activation of TGF-β1/Smad pathway. This activation led to the induction and promotion of EMT in renal tubular epithelial cells and the subsequent onset of TIF. Similarly, this study showed that in NRK52Es treated with high glucose, the expression of E-cadherin (a marker for epithelial cells) was significantly reduced, while the expression of α-SMA (a marker for mesenchymal cells) and FN (an important component of ECM) was significantly increased, the mRNA and protein levels of TGF-β1 was remarkably increased, however the protein level of SnoN decreased dramatically. These results suggested that high glucose induced and promoted EMT by elevating the expression of TGF-β1 and reducing the expression of SnoN. Notably, increased TGF-β1 stimulates SnoN transcription [12, 48]. In our study, high glucose promoted the protein level of SnoN upregulation while the mRNA level downregulation, which implies that the expression of SnoN was regulated at the post-transcriptional level, including protein ubiquitination degradation. Furthermore, we performed in vitro SnoN ubiquitination assay and found that high glucose promoted markedly poly-ubiquitinated of SnoN.

As important regulators, ubiquitin ligases modulate signaling pathways. Arkadia is a member of the RING finger ubiquitin ligase superfamily that promotes activation of the TGF-β1 signaling pathway. Once TGF-β1 signaling is activated, Arkadia binds to p-Smad2/3 [15–17] and induces degradation of the SnoN/Ski repressors [15, 16, 19], enabling transcription of TGF-β target genes. In our study, the expression of Arkadia, p-Smad2 and p-Smad3 significantly increased in NRK52Es treated with high glucose, demonstrating that increased Arkadia promotes the ubiquitin-mediated degradation of SnoN protein mediated by p-Smad2/Smad3.

OM is a traditional Chinese herbal product. As the main active component of matrine alkaloid, OM has multiple pharmacological effects and functions. OM could attenuate liver fibrosis, pulmonary fibrosis, myocardial fibrosis and skin scar tissue fibrosis via inhibiting TGF-β1/Smad signaling pathway. However, it is not known whether OM can attenuate renal fibrosis in the development of DN. Our findings showed that OM could reverse the remarkable decrease of E-cadherin and SnoN and significantly increase α-SMA, FN, TGF-β1, Arkadia, p-Smad2 and p-Smad3 and remarkly attenuate the ubiquitin-dependent degradation of SnoN induced by high glucose, which indicates that OM can inhibit EMT induced by high glucose via inhibiting TGF-β1/Smad signaling pathway by reducing ubiquitin-dependent degradation of SnoN.

## Conclusion

Our results demonstrated that OM can inhibit the high glucose-induced renal tubal EMT by reducing the degradation of SnoN mediated by Arkadia and inhibiting activation of the TGF-β1/Smad signaling pathway. Thus, OM may be an effective therapeutic drug and SnoN may be a potential therapeutic target for the treatment of DN.

## References

[1] Satirapoj B. Nephropathy in diabetes. Advances in Experimental Medicine and Biology. 2012;771:107–122. 23393675 10.1007/978-1-4614-5441-0_11

[2] Sun YM, Su Y, Li J, Wang LF. Recent advances in understanding the biochemical and molecular mechanism of diabetic nephropathy. Biochemical and Biophysical Research Communications. 2013;433(4):359–361. 10.1016/j.bbrc.2013.02.120 23541575

[3] Kolset SO, Reinholt FP, Jenssen T. Diabetic nephropathy and extracellular matrix. Journal of Histochemistry and Cytochemistry. 2012;60(12):976–986. 10.1369/0022155412465073 23103723 PMC3527883

[4] Herbach N. Pathogenesis of diabetes mellitus and diabetic complications. Studies on diabetic mouse models. Der Pathologe. 2012 Suppl 2:318–24. 10.1007/s00292-012-1637-1 23052340

[5] Liu Y. New insights into epithelial-mesenchymal transition in kidney fibrosis. J. Am. Soc. Nephrol. 2010;21(2):212–22. 10.1681/ASN.2008121226 20019167 PMC4554339

[6] Bonventre JV. Can we target tubular damage to prevent renal function decline in diabetes? Seminars in Nephrology. 2012;32(5):452–562. 10.1016/j.semnephrol.2012.07.008 23062986 PMC3595316

[7] Lan HY. Diverse roles of TGF-β/Smad in renal fibrosis and inflammation. Int J Biol Sci. 2011;7:1056–1067. 21927575 10.7150/ijbs.7.1056 PMC3174390

[8] Bottinger EP. TGF-beta in renal injury and disease. Semin Nephrol. 2007;27:309–320. 17533008 10.1016/j.semnephrol.2007.02.009

[9] Hills CE, Squires PE. TGF-beta1-induced epithelial-to-mesenchymal transition and therapeutic intervention in diabetic nephropathy. Am J Nephrol. 2010;31(31):68–74. 19887790 10.1159/000256659

[10] Hills CE, Squires PE. The role of TGF-β and epithelial-to mesenchymal transition in diabetic nephropathy. Cytokine Growth Factor Rev. 2011;22(3):131–139. 10.1016/j.cytogfr.2011.06.002 21757394

[11] Luo K. Ski and SnoN negative regulators of TGF-beta signaling. Curr Opin Genet Dev. 2004;14(1):65–70. 15108807 10.1016/j.gde.2003.11.003

[12] Deheuninck J, Luo K. Ski and SnoN potent negative regulators of TGF-beta signaling. Cell Res. 2009;19(1):47–57. 10.1038/cr.2008.324 19114989 PMC3103856

[13] Tan R, Zhang J, Tan X, Zhang X, Yang J, Liu Y. Downregulation of SnoN expression in obstructive nephropathy is mediated by an enhanced ubiquitin-dependent degradation. Journal of the American Society of Nephrology. 2006;17(10):2781–2791. 16959829 10.1681/ASN.2005101055

[14] Inoue Y, Imamura T. Regulation of TGF-β family signaling by E3 ubiquitin ligases. Cancer Science. 2008;99(11):2107–2112. 10.1111/j.1349-7006.2008.00925.x 18808420 PMC11158544

[15] Le Scolan E, Zhu Q, Wang L, Bandyopadhyay A, Javelaud D, Mauviel A, et al Transforming growth factor-beta suppresses the ability of Ski to inhibit tumor metastasis by inducing its degradation. Cancer Res. 2008;68(9):3277–3285. 10.1158/0008-5472.CAN-07-6793 18451154

[16] Levy L, Howell M, Das D, Harkin S, Episkopou V, Hill CS. Arkadia activates Smad3/Smad4-dependent transcription by triggering signal-induced SnoN degradation. Mol. Cell. Biol. 2007;27(17):6068–6083. 17591695 10.1128/MCB.00664-07 PMC1952153

[17] Mavrakis KJ, Andrew RL, Lee KL, Petropoulou C, Dixon JE, Navaratnam N, et al Arkadia enhances Nodal/TGF-beta signaling by coupling phospho-Smad2/3 activity and turnover. PLoS Biol. 2007;5(3):e67 17341133 10.1371/journal.pbio.0050067 PMC1808117

[18] Koinuma D, Shinozaki M, Komuro A, Goto K, Saitoh M, Hanyu A, et al Arkadia amplifies TGF-beta superfamily signalling through degradation of Smad7. EMBO J. 2003; 22(24):6458–6470. 14657019 10.1093/emboj/cdg632 PMC291827

[19] Nagano Y, Mavrakis KJ, Lee KL, Fujii T, Koinuma D, Sase H, et al adia induces degradation of SnoN and c-Ski to enhance transforming growth factor-beta signaling.J. Biol. Chem. 2007;282(28):20492–20501. 17510063 10.1074/jbc.M701294200

[20] Cao CX, Yang QW, Lv FL, Cui J, Fu HB, Wang JZ. Reduced cerebral ischemia-reperfusion injury in Toll-like receptor 4 deficient mice. Biochemical and Biophysical Research Communications.2007;353(2):509–514. 17188246 10.1016/j.bbrc.2006.12.057

[21] Lu LG, Zeng MD, Mao YM, Li JQ, Wan MB, Li CZ, et al Oxymatrine therapy for chronic hepatitis B: a randomized double-blind and placebo-controlled multi-center trial. World Journal of Gastroenterology.2003;9(11):2480–83. 14606080 10.3748/wjg.v9.i11.2480 PMC4656524

[22] Wang YP, Zhao W, Xue R, Zhou ZX, Liu F, Han YX, et al Oxymatrine inhibits hepatitis B infection with an advantage of overcoming drug-resistance. Antiviral Research. 2011;89(3):227–231. 10.1016/j.antiviral.2011.01.005 21277330

[23] Deng ZY, Li J, Jin Y, Chen XL, Lü XW. Effect of oxymatrine on the p38 mitogen-activated protein kinases signalling pathway in rats with CCl4 induced hepatic fibrosis. Chin. Med. J. 2009;122(12):1449–1454. 19567170

[24] Shi GF, Li Q. Effects of oxymatrine on experimental hepatic fibrosis and its mechanism in vivo. World J Gastroenterol. 2005;11(2):268–271. 15633229 10.3748/wjg.v11.i2.268 PMC4205415

[25] Yu XH, Zhu JS, Yu HF, Zhu L. Immunomodulatory effect of oxymatrine on induced CCl4-hepatic fibrosis in rats. Chin Med J. 2004;117(12):1856–1858. 15603720

[26] Liu L, Lu W, Ma Z, Li Z. Oxymatrine attenuates bleomycin-induced pulmonary fibrosis in mice via the inhibition of inducible nitric oxide synthase expression and the TGF-β/Smad signaling pathway. Int J Mol Med. 2012; 29(5):815–822. 10.3892/ijmm.2012.923 22367596

[27] Wu X, Zeng WZ, Jiang MD, Qin JP, Xu H. Effect of Oxymatrine on the TGF beta-Smad signaling pathway in rats with CCl4-induced hepatic fibrosis. World J Gastroenterol. 2008;14(13):2100–2105. 18395914 10.3748/wjg.14.2100 PMC2701534

[28] Shi GF, Li Q. Effects of oxymatrine on experimental hepatic fibrosis and its mechanism in vivo. World J Gastroenterol. 2005;11(2):268–271. 15633229 10.3748/wjg.v11.i2.268 PMC4205415

[29] Chen X, Sun R, Hu J, Mo Z, Yang Z, Liao D, et al Attenuation of Bleomycin-Induced Lung Fibrosis by Oxymatrine Is Associated with Regulation of Fibroblast Proliferation and Collagen Production in Primary Culture. Basic Clin Pharmacol Toxicol. 2008;103(3):278–286. 10.1111/j.1742-7843.2008.00287.x 18684219

[30] Shen XC, Yang YP, Xiao TT, Peng J, Liu XD. Protective effect of oxymatrine on myocardial fibrosis induced by acute myocardial infarction in rats involved in TGF-β1-Smad signal pathway. J Asian Nat Prod Res. 2011;13(3):215–224. 10.1080/10286020.2010.550883 21409682

[31] Fan DL, Zhao WJ, Wang YX, Han SY, Guo S. Oxymatrine inhibits collagen synthesis in keloid fibroblasts via inhibition of transforming growth factor-β1/Smad signaling pathway. Int J Dermatol. 2012;51(4):463–472. 10.1111/j.1365-4632.2011.05234.x 22435440

[32] Tang F, Wang B, Li N, Wu YF, Jia JY, Suo TL, et al RNF185, a Novel Mitochondrial Ubiquitin E3 Ligase, Regulates Autophagy through Interaction with BNIP1. PLoS One. 2011; 6(9): e24367 10.1371/journal.pone.0024367 21931693 PMC3170314

[33] Togawa A, Yamamoto T, Suzuki H, Fukasawa H, Ohashi N, Fujigaki Y, et al Ubiquitin-dependent degradation of Smad2 is increased in the glomeruli of rats with anti-thymocyte serum nephritis. Am J Pathol. 2003;163(4): 1645–1652. 14507671 10.1016/S0002-9440(10)63521-3 PMC1868293

[34] Fukasawa H, Yamamoto T, Togawa A, Ohashi N, Fujigaki Y, Oda T, et al Down-regulation of Smad7 expression by ubiquitin-dependent degradation contributes to renal fibrosis in obstructive nephropathy in mice. Proc Natl Acad Sci U S A. 2004; 101(23): 8687–8692. 15173588 10.1073/pnas.0400035101 PMC423256

[35] Liu LR, Li S, Wang YY, Shi MJ, Xiao Y, Guo B. Oxymatrinein Inhibits the Renal Tubal EMT Induced by High Glucose and Its Possible Mechanism. Chinese Journal of Pathophysiology. 2013;29(12):2152–2159.

[36] Mason RM, Wahab NA. Extracellular matrix metabolism in diabetic nephropathy. J Am Soc Nephrol. 2003;14(5):1358–1373. 12707406 10.1097/01.asn.0000065640.77499.d7

[37] Liu R, Wang Y, Xiao Y, Shi M, Zhang G, Guo B. SnoN as a Key Regulator of the High Glucose-Induced Epithelial-Mesenchymal Transition in Cells of the Proximal Tubule. Kidney Blood Press Res. 2012;35(6):517–528. 10.1159/000339172 22813962

[38] Briones-Orta MA, Levy L, Madsen CD, Das D, Erker Y, Sahai E, et al Arkadia regulates tumor metastasis by modulation of the TGF-β pathway. Cancer Research. 2013;73(6):1800–1810. 10.1158/0008-5472.CAN-12-1916 23467611 PMC3672972

[39] Miyazono K, Koinuma D. Arkadia-beyond the TGF-β pathway. Journal of Biochemistry. 2011;149(1):1–3. 10.1093/jb/mvq133 21109559

[40] Moustakas A, Heldin CH. Coordination of TGF-β signaling by ubiquitylation. Molecular Cell.2013;51(5):555–556. 10.1016/j.molcel.2013.08.034 24034692

[41] Hills CE, Squires PE. TGF-beta1-induced epithelial-to-mesenchymal transition and therapeutic intervention in diabetic nephropathy. The American Journal of Nephrology. 2009;31(1):68–74. 10.1159/000256659 19887790

[42] Zhou X, Wang B, Zhu L, Hao S. A novel improved therapy strategy for diabetic nephropathy: targeting AGEs. Organogenesis. 2012;8(1):18–21. 10.4161/org.19332 22349714 PMC3399706

[43] Briones-Orta MA, Sosa-Garrocho M, Moreno-Alvarez P, Fonseca-Sánchez MA, Macías-Silva M. SnoN co-repressor binds and represses SnoN gene promoter.Biochem Biophys Res Commun. 2006;341(3):889–894. 16442497 10.1016/j.bbrc.2006.01.041

[44] Krakowski AR, Laboureau J, Mauviel A, Bissell MJ, Luo K. Cytoplasmic SnoN in normal tissues and nonmalignant cells antagonizes TGF-beta signaling by sequestration of the Smad proteins. Proc Natl Acad Sci USA. 2005;102(35):12437–12442. 16109768 10.1073/pnas.0504107102 PMC1194926

[45] Javelaud D, van Kempen L, Alexaki VI, Le Scolan E, Luo K, Mauviel A. Efficient TGF-β/SMAD signaling in human melanoma cells associated with high c-SKI/SnoN expression. Mol Cancer. 2011;10(1):2 10.1186/1476-4598-10-2 21211030 PMC3025974

[46] Fujita H, Omori S, Ishikura K, Hida M, Awazu M. M. ERK and p38 mediate high-glucose-induced hypertrophy and TGF-beta expression in renal tubular cells. Am J Physiol Renal Physiol. 2004;286(1):F120–126. 12952860 10.1152/ajprenal.00351.2002

[47] Gu L, Gao Q, Ni L, Wang M, Shen F. Fasudil Inhibits Epithelial-Myofibroblast Transdifferentiation of Human Renal Tubular Epithelial HK-2 CellsInduced by High Glucose. Chem Pharm Bull (Tokyo). 2013;61(7):688–694. 23812394 10.1248/cpb.c13-00066

[48] Pot I, Bonni S. SnoN in TGF-beta signaling and cancer biology. Curr Mol Med. 2008;8(4):319–328. 18537639 10.2174/156652408784533797

